# Direct‑vision peritoneal access as salvage for guidewire loss during endoscopic ultrasound‑guided hepaticojejunostomy

**DOI:** 10.1055/a-2906-8199

**Published:** 2026-07-23

**Authors:** Ke Han, Haoran Li, Yang Liu, Nana Guo, Yong Sun, Feng Gao, Zhuo Yang

**Affiliations:** 1 Department of Endoscopy 74643 General Hospital of Northern Theatre CommandShenyangLiaoningChina; 2 Department of GastroenterologyAffiliated Zhongshan Hospital Dalian UniversityDalianLiaoningChina

**Fig. 1:**
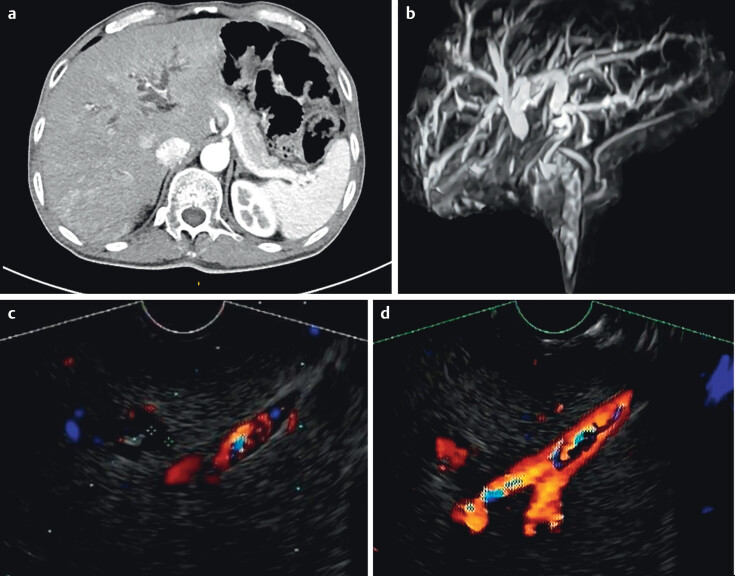
Preprocedural imaging and EUS findings. ( **a** ) Contrast-enhanced computed tomography showing dilatation of the intrahepatic bile ducts. ( **b** ) Magnetic resonance cholangiopancreatography showing upstream intrahepatic duct dilatation with an abrupt hilar biliary stricture. ( **c** ) EUS from the jejunal limb showing a mildly dilated left intrahepatic duct (a diameter of approximately 3.3 mm). ( **d** ) EUS after guidewire loss showing collapse of the left intrahepatic ducts.

A 60-year-old man with a history of total gastrectomy with Roux-en-Y reconstruction and cholecystectomy presented with progressive jaundice. Computed tomography and magnetic resonance cholangiopancreatography showed a hilar biliary stricture with upstream intrahepatic duct dilatation, suspicious for malignancy ( Fig. 1a, b ). Enteroscopy-assisted endoscopic retrograde cholangiopancreatography failed because a guidewire could not traverse the complete hilar obstruction. The patient declined surgical and percutaneous drainage.

Endoscopic ultrasound (EUS)-guided hepaticojejunostomy was therefore attempted from the jejunal limb. 1​ ​2​ The segment II duct (3.3 mm) was punctured with a 19-gauge needle, and a guidewire was advanced but remained above the hilar stricture. After tract dilation with a 10-Fr cystotome, the guidewire was accidentally lost during device exchange. Immediate bile leakage caused decompression and collapse of the intrahepatic ducts, precluding EUS-guided repuncture ( Fig. 1c, d ).

**Fig. 2:**
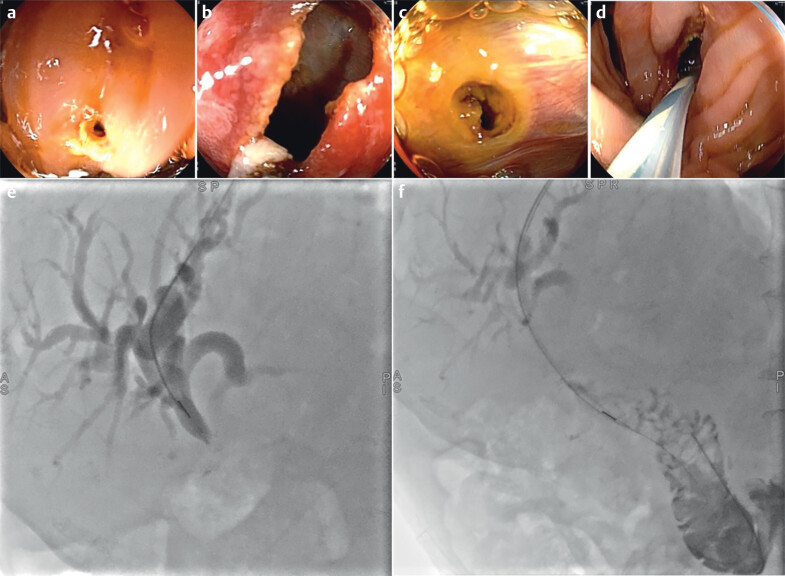
Endoscopic and fluoroscopic salvage procedures. ( **a** ) Endoscopic identification of the jejunal puncture site. ( **b** ) Controlled enlargement of the jejunal opening with a needle-knife. ( **c** ) A direct endoscopic view of the bile-leaking hepatic puncture site on the liver surface. ( **d** ) Recannulation of the hepatic puncture site using a sphincterotome loaded with a guidewire. ( **e** ) Fluoroscopic cholangiography after re-entry into the biliary system, showing opacification of the intrahepatic ducts with abrupt hilar cutoff. ( **f** ) A fluoroscopic image showing successful guidewire passage across the hilar stricture with contrast flow into the small-bowel lumen.

**Fig. 3:**
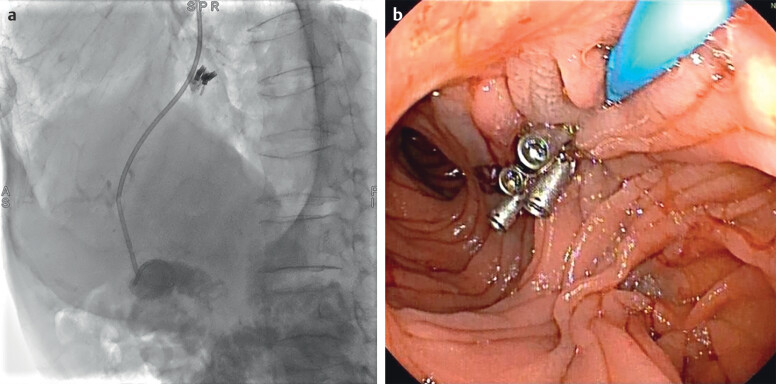
Stent placement and defect closure. ( **a** ) A fluoroscopic image showing the placement of a nasobiliary catheter across the hepaticojejunal tract. ( **b** ) An endoscopic view showing complete closure of the jejunal defect with endoclips.

As salvage, a forward-viewing gastroscope was advanced to the jejunal puncture site, and the opening was cautiously enlarged with a needle-knife to allow entry into the subhepatic peritoneal cavity ( Video 1 ). Under direct endoscopic visualization, the bile-leaking hepatic puncture site was identified on the liver surface ( Fig. 2a–c ). A sphincterotome preloaded with a guidewire was introduced into this defect, and fluoroscopy confirmed intraductal access. After repeated manipulation, the guidewire was advanced across the hilar stricture into the small-bowel lumen ( Fig. 2d–f ). The tract was then dilated with a balloon catheter, and a 7-Fr pigtail nasobiliary catheter was placed across the hepaticojejunal tract with its distal end in the small-bowel lumen. The jejunal defect was closed with endoclips ( Fig. 3 ).

**Video 1** Endoscopic rescue for EUS-HJS guidewire loss.


The patient received antibiotics and recovered without peritonitis. On day 4, the external portion of the nasobiliary catheter was cut endoscopically to internalize the stent. No abdominal pain, fever, or recurrent jaundice developed during 6 weeks of follow-up. Direct-vision peritoneal access may offer a rescue option after guidewire loss during EUS-guided biliary drainage when conventional re-access fails in expert hands. 3​ ​4​

Endoscopy_UCTN_Code_TTT_1AO_2AO
